# Advancements in mesenchymal stem cell therapy for chronic wounds: challenges, innovations, and future directions

**DOI:** 10.3389/fcell.2025.1730032

**Published:** 2026-01-12

**Authors:** Yi Tang, Tong Su, Baochuan Huang, Qihong Xu, Qingjiang Chen, Saiqiong Zhang, Wuquan Li

**Affiliations:** Department of Burn Surgery, The Second Affiliated Hospital of Kunming Medical University, Kunming, China

**Keywords:** angiogenesis, chronic wounds, immune modulation, mesenchymal stem cells, paracrine signaling, regenerative medicine

## Abstract

The management of chronic wounds remains challenging due to their complex pathophysiology, poor response to conventional therapies, and significant impact on patients’ quality of life. mesenchymal stem cells (MSCs) have garnered attention as a potential treatment due to their regenerative and immunomodulatory properties. This review summarizes preclinical and clinical advancements in MSC-based therapies for chronic wound healing. MSCs promote tissue regeneration through various mechanisms, including differentiation into skin cell lineages, modulation of inflammation, angiogenesis, and paracrine release of bioactive factors. Current research focuses on identifying viable MSC sources, optimizing delivery methods, and understanding their mechanisms for clinical use. Despite progress, challenges remain, including inconsistent results, poor MSC survival in the wound microenvironment, and variability in regenerative capacity across MSC sources. Future research should focus on developing standardized guidelines for MSC preparation and conducting long-term randomized trials to assess safety, efficacy, and potential risks. In conclusion, this review highlights current evidence and identifies key challenges for the clinical application of MSCs in chronic wound healing.

## 1 Introduction

### 1.1 Definition and clinical challenges of chronic wounds

Chronic wounds are defined as skin or soft tissue injuries that fail to progress through the normal stages of healing within a typical timeframe, generally persisting for more than 3 months (Li and Zheng, 2024). Examples of chronic wounds include diabetic foot ulcers (DFUs), pressure ulcers (PUs), and venous leg ulcers (VLUs) as well as iatrogenic lesions such as radiation-induced ulcers that develop after radiotherapy These wounds exhibit a high incidence and recurrence rate and are often refractory to treatment (Li et al., 2023; Liu et al., 2024; Jones et al., 2018). The pathophysiology of chronic wounds is complex and multifactorial, involving factors such as chronic inflammation, hypoxia, infection, vascular insufficiency and cellular dysfunction. Although chronic wounds share several core pathological features, different etiological subtypes—such as neuropathic DFUs, ischemia- and pressure-driven ulcers and radiation- induced ulcers characterized by microvascular rarefaction and fibrosis—arise from distinct combinations of these mechanisms. These elements are critical to the healing process, and impaired healing results in prolonged suffering, significantly diminishing the patient’s quality of life (Abbott et al., 2019; Li et al., 2021; Schilrreff and Alexiev, 2022).

Chronic wounds represent a substantial global healthcare burden. Epidemiological studies estimate that 19%–34% of diabetic patients will develop DFUs, with approximately 15% experiencing at least one ulceration during their disease course (Rastogi et al., 2020). The prevalence of PUs ranges from 2.5% to 15% among hospitalized patients, and up to 30% in long-term care facilities (Tomova-Simitchieva et al., 2019). VLUs affect 1%–2% of the general population, with a higher prevalence among the elderly (Probst et al., 2023). As survival rates for cancer patients continue to improve, the prevalence of chronic wounds caused by radiation therapy (burns) will grow along with this increase in cancer patient survivors. These will create an additional burden of care for patients previously treated with radiation therapy as the incidence of chronic wounds from radiation continues to grow. The economic burden of chronic wounds is significant, with the annual direct cost of DFU management in the United States estimated at USD 9.7 billion, and an additional USD 9 billion spent on PU management (Sa et al., 2024; Kuikko et al., 2024). Beyond the financial costs, patients endure severe pain, functional impairment, infection risk, and psychosocial distress, with advanced DFUs leading to a high risk of lower-limb amputation—only 50% of which result in survival beyond 5 years (Schreml and Berneburg, 2017; Hicks et al., 2019).

Standard treatments, including negative pressure wound therapy (NPWT), growth factor therapy, and surgery, offer temporary relief at best (de Oliveira et al., 2018). However, they fail to address the underlying pathological imbalances within the wound microenvironment, and these interventions do not effectively reverse chronic inflammation, promote angiogenesis, or restore cellular function. This limitation is found in all types of chronic wounds, but is especially noticeable in the formation of iatrogenic lesions, including those caused by radiotherapy-induced ulceration, which in many cases, severely limit the ability of reconstructive techniques to restore tissues to normal function due to the presence of extensive microvascular damage and extensive fibrosis. Consequently, there is a pressing need to develop new therapies that focus on healing the wound rather than merely managing symptoms (Oe et al., 2022). The limitations of current treatments highlight the need for more effective approaches, such as:

- Negative pressure wound therapy (NPWT): Improves local perfusion and granulation tissue formation but does not resolve chronic inflammation or cellular senescence (Takagi et al., 2020; Norman et al., 2022).
- Growth factor therapy: Agents such as epidermal growth factor (EGF) and platelet-derived growth factor (PDGF) stimulate cell proliferation, yet their short half-lives and rapid degradation within inflamed tissue limit clinical benefit (Rogers and Fantauzzo, 2020; Wang et al., 2019).
- Surgical repair: Skin grafting or reconstructive procedures may be performed, but outcomes are suboptimal when infection or impaired microcirculation or extensive radiation-induced fibrosis is present (Ukai et al., 2020).
- Conventional dressings and antibiotics: Provide supportive care but do not modulate the underlying pathophysiology (Vivcharenko et al., 2023; Long et al., 2022).

**FIGURE 1:**
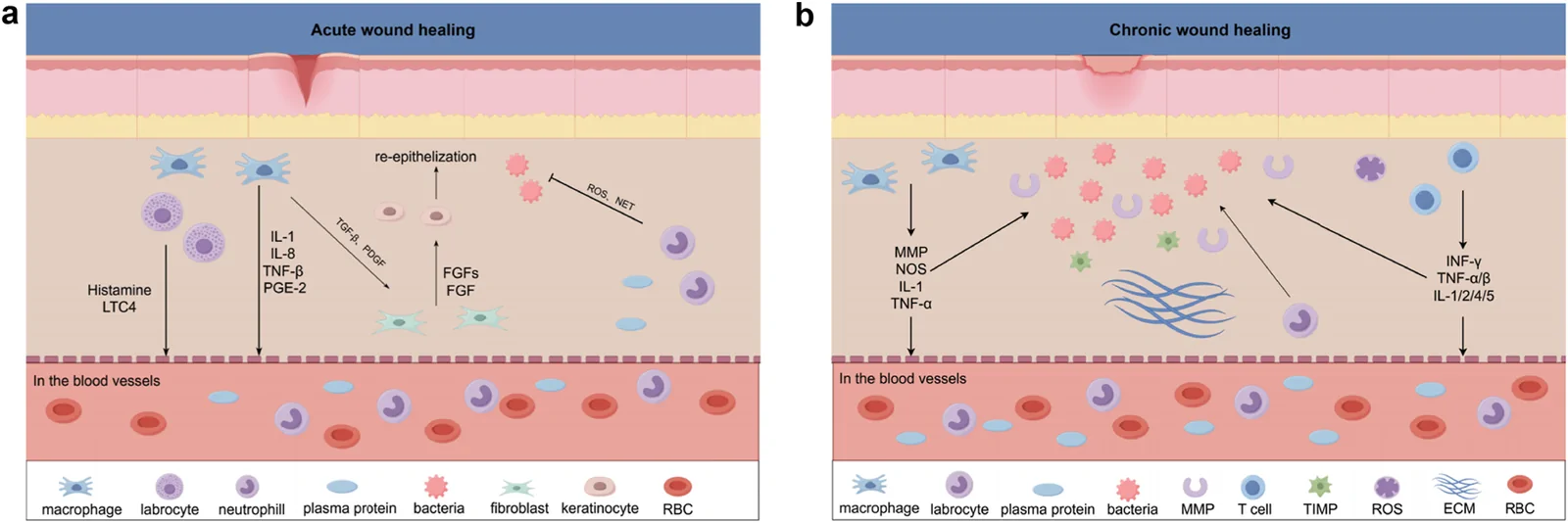
**(a)** Acute Wound Healing In acute wound healing, local immune responses promote inflammation and re-epithelialization through the release of cytokines such as IL-1, IL-8, TNF-β, and PGE-2. Factors like Histamine and LTC4 further enhance the local immune response, while TGF-β and FGF play essential roles in re-epithelialization. Neutrophils and macrophages in the blood vessels are recruited to the wound site, aiding in the clearance of bacteria and dead cells. **(b)** Chronic Wound Healing In chronic wound healing, persistent inflammation impedes the normal healing process, with factors such as MMP, NOS, IL-1, and TNF-α maintaining prolonged local inflammation. Unlike acute wounds, angiogenesis is impaired in chronic wounds. INF-γ, TNF-α/β, and IL-1/2/4/5 are released, modulating immune responses and influencing various stages of wound healing. MSCs play a critical role in this process through immune modulation, promoting angiogenesis, and paracrine effects, offering potential therapeutic approaches.

These differences between acute and chronic wound healing are summarized in Figure 1, highlighting the pathological barriers that MSC-based therapies are uniquely positioned to overcome. Given these challenges, MSC-based therapies, which address multiple aspects of the wound-healing process, are best conceptualized as adjuncts or biological intensifiers of conventional treatments rather than stand-alone alternatives. When added on top of standard wound care—including debridement, infection control, off- loading, revascularization and advanced dressings—MSCs may exert complementary and even synergistic effects on the chronic wound microenvironment. strategies and are likely to be used in combination with, rather than as a replacement for, conventional treatments. The primary objectives in chronic wound management are to accelerate tissue repair, control infection, and enhance quality of life (Savelieff et al., 2025). Nevertheless, conventional therapies remain insufficient because they fail to correct the pathological microenvironment.

### 1.2 Basic concepts of MSCs and rationale for chronic wound therapy

Mesenchymal stem cells (MSCs) are multipotent adult progenitor cells that were first described in bone marrow and have since been identified in adipose tissue, umbilical cord, placenta and many other sources (Dipalma et al., 2025; Daltro et al., 2020; AJ, 1968). The three main properties of MSCs are multi-lineage differentiation, immunomodulation, and paracrine signaling, which define their therapeutic warrant in regenerative medicine (Kobolak et al., 2016; Lelek and Zuba-Surma, 2020).

Chronic wounds such as diabetic foot ulcers, pressure ulcers, and venous leg ulcers have specific characteristics of chronic inflammation, ineffective angiogenesis, and aberrant extracellular matrix remodeling, which serves as obstacles to healing (Kouroupis et al., 2023). The employment of MSCs provides a counter to these chronic wound characteristics. MSCs mitigate chronic inflammation by secreting anti-inflammatory cytokines (i.e., IL-10, TGF-β) and by modulating immune cell populations (Azevedo et al., 2020; Darlan et al., 2021; Aulia et al., 2024). MSCs will further mediate chronic wound repair through paracrine secretion of growth factors, such as VEGF, HGF, and bFGF, which promote angiogenesis in addition to cell proliferation and survival to improve the formation and re-epithelialization of granulation tissue. Although MSCs can differentiate into fibroblasts, endothelial cells, and keratinocytes, this differentiation process is controversial and is not definitively proven. Current models of chronic wound healing emphasize primarily the paracrine and immunomodulatory properties of MSCs as the mechanisms responsible for effecting healing (Cao et al., 2017; Legrand and Martino, 2022; Fernandes et al., 2020; Garikipati et al., 2018; Akasaka, 2022; Kieper et al., 2010; Fang et al., 2020).

At the molecular level, MSCs act mainly via paracrine signalling and extracellular vesicles (EVs) to recalibrate chronic wounds: they resolve inflammation by downregulating NF-κB/NLRP3 and skew macrophages towards M2 via IL-10/TGF-β signalling (Al-Shaibani et al., 2024); promote angiogenesis through the HIF-1α → VEGF axis and pro-angiogenic miRNAs (e.g., miR-126/miR-210) (Pea and Martin, 2024); accelerate re-epithelialisation via EGFR/ERK and Wnt/β-catenin cues (Mamun et al., 2024); and normalise ECM turnover via TGF-β/Smad with a balanced MMP/TIMP programme. These pathway-level effects provide the mechanistic basis for the improvements observed in preclinical and early clinical studies.

Preclinical studies demonstrate that MSCs restore wound homeostasis and facilitate closure, and early phase clinical trials are now confirming their potential as a therapeutic modality (Kouroupis et al., 2023). Regardless, substantial translational barriers exist, including source heterogeneity, variability from donor to donor with respect to immunomodulatory capacity, variability in manufacturing processes, variability in delivery approaches, and limited long-term safety data (Volarevic et al., 2018; Maldonado et al., 2023; Chen and Liu, 2016). If these translational barriers can be overcome, with particular reference to potency assays that can be standardized, along with rational clinical guidelines, MSCs can take their place as reliable, reproducible and clinically useful acute or chronic wound therapies.

### 1.3 Objectives and significance of the study

**FIGURE 2:**
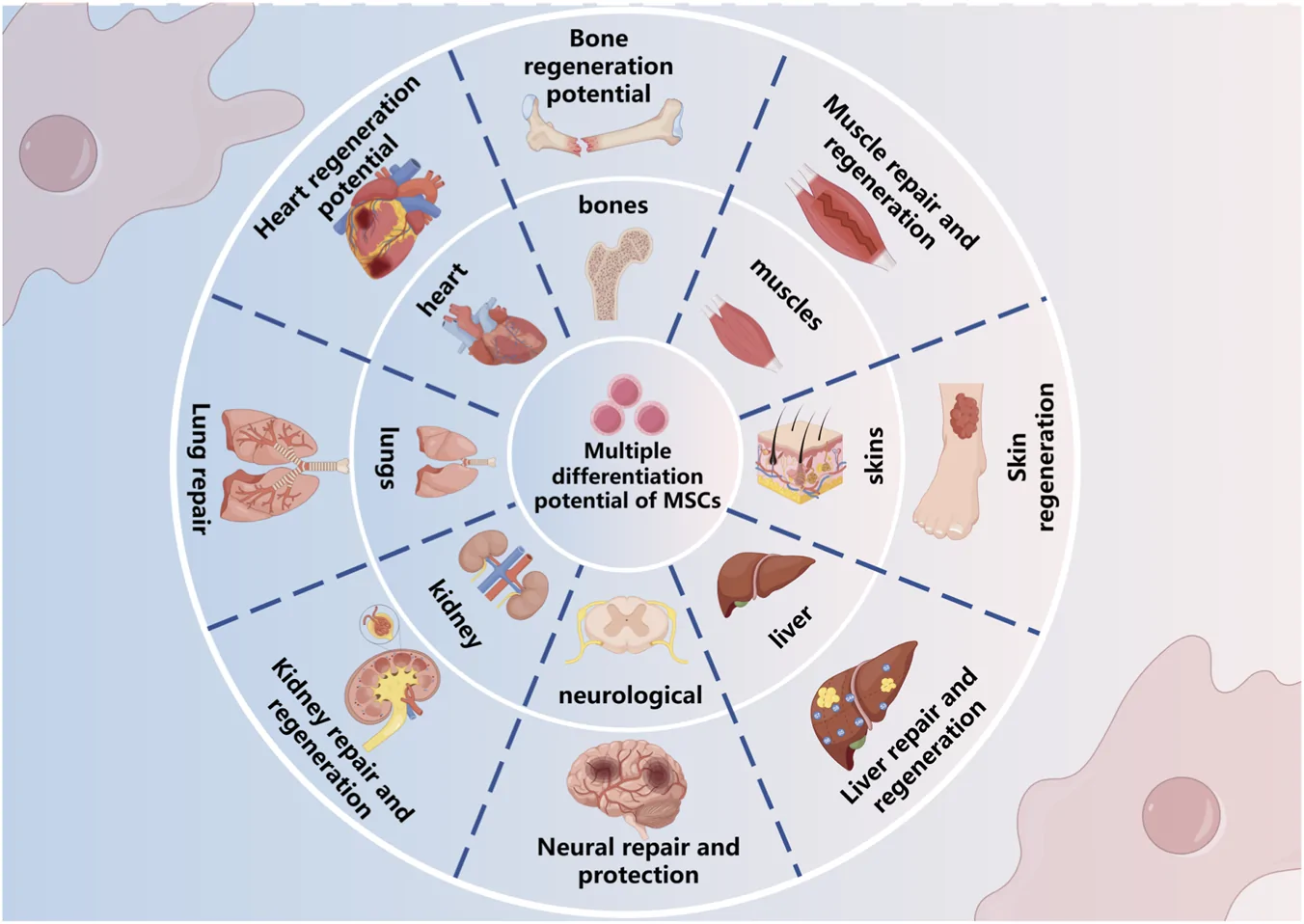
Multiple differentiation potential of mesenchymal stem cells (MSCs). MSCs possess the capacity to differentiate into various tissue types, including bone, muscle, skin, liver, kidney, lung, heart, and nervous system, contributing to tissue repair and regeneration across multiple organ systems.

This review aims to comprehend worldwide studies related to the use of mesenchymal stem cells (MSCs) to manage chronic wounds, focusing on three principal domains: (i) the biological effects of MSCs related to differentiation, immune modulation, and paracrine activity; (ii) clinical studies of MSCs from preclinical and clinical data; and (iii) the current obstacles to the translational uptake of MSC therapies into clinical practice. This review seeks to summarize the research evidence to date that relates MSC activity to tissue repair, illuminate key considerations for future research, and review important gaps in the evidence needed to advance MSC therapies into a clinical setting. The impact of MSC therapies on patients clinically and societally is significant, potentially improving the quality of life for patients with acute or chronic wounds, while simultaneously reducing the economic burden chronic wound care places on the healthcare system, should MSC therapy prove effective. Thus, even should MSC therapies be successful and clinical, substantial obstacles still remain to enable the full potential of this therapy including efficacy due to sample source heterogeneity, safety concerns, and developing treatment protocols and standardized processes. Ultimately, successful MSC therapies in a clinical setting could be a game-changer for managing chronic wounds and has the potential to broaden to other therapeutic areas in regenerative medicine (Figure 2).

## 2 Mechanism of action of mesenchymal stem cells in the treatment of chronic wounds

### 2.1 Paracrine action

Mesenchymal stem cells (MSCs) promote wound healing primarily by secreting bioactive molecules, particularly key growth factors involved in angiogenesis, cellular proliferation, and extracellular matrix (ECM) remodeling (Harrell et al., 2019b; Legrand and Martino, 2022). In addition to soluble factors, extracellular vesicles, including exosomes, also act as important carriers of these paracrine signals and will be discussed in more detail in Section 2.4. Vascular endothelial growth factor (VEGF) and fibroblast growth factors (FGFs) are key regulators of angiogenesis by promoting endothelial cellular proliferation and migration, as well as lumen formation via phosphoinositide 3-kinase (PI3K)/protein kinase B (AKT) and extracellular signal-regulated kinase (ERK)/mitogen-activated protein kinases (MAPK) signaling pathways (Wang et al., 2019; Kasowanjete et al., 2024). Beyond driving the core processes of angiogenesis, VEGF increases vascular permeability, thereby facilitating the recruitment of endothelial progenitor cells to the injury site, whereas FGFs upregulate the production of matrix metalloproteinases (MMPs), which are essential for ECM remodeling and for the ingrowth of new blood vessels (Sung et al., 2021). Cytokines derived from MSCs, such as interleukin-6 (IL-6) and IL-8, aid the angiogenic process by promoting the expression of VEGF and FGF which, in turn, amplifies the overall healing cascade (Harrell et al., 2019b; Fernandes et al., 2020). In addition to angiogenesis, MSCs also secrete epidermal growth factor (EGF) and keratinocyte growth factor (KGF) which stimulate keratinocyte proliferation, migration, and differentiation via the EGF receptor (EGFR) and FGF receptor 2b (FGFR2b) respectively (Zhang et al., 2023). Transforming growth factor-β (TGF-β) derived from MSCs promotes fibroblast differentiation and drives the production of collagen and other ECM components that provide structural support during epithelial barrier repair (Akasaka, 2022). Collectively, these paracrine signaling mechanisms create a localized environment that supports angiogenesis and simultaneously sustains dynamic epithelial repair. Some of these mediators also contribute to immunomodulation, which is further elaborated in Section 2.2. While the paracrine effects of MSCs are clearly beneficial, recent studies suggest that successful wound repair relies on the coordinated activation of multiple signaling pathways rather than isolated mechanisms (Zhang et al., 2024). Moreover, this new potential has led researchers to explore the genetic modification of MSCs and to enhance bioactive molecules in exosomes. However, we are still faced with many challenges, especially the dynamics, stability, reproducibility, and therapeutic efficiency of these bioactive molecules when considering the clinical setting (Yang Q. et al., 2022). We still have to address many of the issues faced in current clinical practice with not only the standardization of MSC-based therapy but also with the potential mechanisms of delivery for consistent control of delivering bioactive molecules (Maldonado et al., 2023). Therefore, the establishment of standardization and controlled procedural mechanism will be paramount for the clinical success and usefulness of cell-based (MSCs) therapy in clinical care.

### 2.2 Immunomodulatory effect

As outlined in Section 2.1, the broad repertoire of soluble factors and extracellular vesicles released by MSCs forms the basis of their immunomodulatory capacity. By acting on both innate and adaptive immune cells, these paracrine mediators play a key role in dampening chronic inflammation that can arise during impaired tissue repair. MSCs secrete a wide variety of compounds that modulate the immune response, including prostaglandins, interleukins, and transforming growth factor-beta. These together establish an anti-inflammatory state and assist in shifting macrophage populations from pro-inflammatory M1 to reparative M2 macrophages (Azevedo et al., 2020; Ong and Dilley, 2018). In addition, MSC- derived exosomes contain microRNAs (miRNAs), such as miR-21 and miR-146a, that exert pro-reparative effects on macrophage polarization by enhancing macrophage function through metabolic reprogramming (Chen et al., 2022). In terms of adaptive immunity, mesenchymal stem cells (MSCs) enhance both generation and expansion through the production of IL-10 and TGF-β to produce Tregs while decreasing the number of activated effector T cells (such as called Teff) and balancing out subtypes of Th1/Th2 with Th17/Treg (Lee et al., 2016). Through their exosomes, MSCs inhibit the activation of the NF-κB signalling cascade and other pro-inflammatory pathways in Tregs during their maturation process, thus creating an immunosuppressive environment (Chen et al., 2024). The effects of the differentiation of Tregs involve an interaction between M2-macrophages and Tregs, with M2-macrophages secreting IL-10 and TGF-β to promote the differentiation of Tregs, creating a further enhancement of the anti-inflammatory environment that they produce (Ivanovski et al., 2024). Due to the combined paracrine-mediated immunomodulatory effects of MSCs, there has been significant interest in utilizing MSC therapy for chronic wounds as well as other immunological diseases. Despite the increased interest surrounding the potential use of MSCs for regenerative medicine, there remain numerous challenges associated with MSC therapies that impede their transition from bench to bedside. These challenges include the variability in MSC characteristics and immunosuppressive activities based upon tissue source, the potential risks associated with excessive/non- specific immunosuppression, and the lack of standardized assays to assess MSC potency. In order to achieve the maximum potential level of safety and effectiveness of MSC therapies, addressing these issues is critical (Table 1).

**TABLE 1:** Mechanisms of MSC-based therapy in wound healing.

Mechanism	Description	Factors/pathways	References	Expected outcomes in wound healing
Paracrine effect	MSCs release factors that enhance tissue regeneration	VEGF, FGF, TGF-*β*, HGF, IL-6	Li and Zheng (2024), Cao et al. (2017)	Promotes tissue regeneration, reduces inflammation, and increases collage
Immune modulation	MSCs modulate immunity to alleviate chronic inflammation	IL-10, TGF-*β*, TNF-*α*	Schilrreff and Alexiev (2022), de Oliveira et al. (2018)	Decreases inflammation and speeds up healing
Angiogenesis	MSCs enhance blood flow by promoting angiogenesis	VEGF, Ang-1, Ang-2, FGF	Jones et al. (2018), Li et al. (2021)	Boosts healing by improving supply
Differentiation into target cells	MSCs differentiate into tissue-specific cell types	Osteoblasts, chondrocytes, adipocytes	Jones et al. (2018), Kobolak et al. (2016)	Drives tissue regeneration and recovery

### 2.3 Cells are directly involved in tissue repair

While it is likely that the predominant mechanisms of mesenchymal stem cells (MSCs)-mediated wound repair is through paracrine signaling and immunomodulation, it is possible that MSCs may contribute through their own differentiation into tissue specific lineages. In chronic wound settings, MSCs have demonstrated transition into fibroblasts, enhancing extracellular matrix (ECM) production and collagen deposition, with increased wound contraction (Kieper et al., 2010). In some cases, MSCs have demonstrated keratinocyte-like phenotypes, contributing to epidermal regeneration and re-epithelialization (Yoo et al., 2025). Regulation of lineage transitions seems to occur via some combination of growth factors and cytokines and their interactions with the ECM, resulting in local organization of the wound microenvironment- to varying degrees (Xu et al., 2024). While research has shown that direct differentiation is possible, the clinical relevance is questioned. Evidence suggests that while the frequency and stability of lineage commitment is important, it is dwarfed by the strong effects of direct paracrine signaling (Casado-Daz et al., 2020). Moreover, variability amongst donor cell type, culture conditions and local wound environments all remained unpredictable and reproducible (Chen et al., 2024). Therefore, while differentiation may offer structural support for repair, caution must be exercised in the interpretation of evidence to support a meaningful contribution. Future studies of MSCs should consider studies optimizing differentiation protocols, improving the microenvironmental cues supporting lineage specificity, and developing reliable *in vivo* tracking of MSC-derived cells. This will ultimately help decide if differentiation should be considered a clinically relevant mechanism, or merely a potential secondary source of benefit in addition to paracrine signaling and immunomodulatory functions (Volarevic et al., 2018).

### 2.4 Synergy with exosomes

As an integral component of the MSC secretome described above, exosomes produced by mesenchymal stem cells (MSC-Exos) have emerged as important contributors to paracrine signaling. They serve as nanoscale transport vehicles that deliver proteins, lipids and nucleic acids to target cells, thereby modulating angiogenesis, innate immune responses and epithelialization. MSC-exosomes are vesicles produced by mesenchymal stem cells (MSCs) that act as transporters of proteins, lipids and nucleic acids. They play a role in paracrine signaling between cells within tissues or organs. They contribute to the process of angiogenesis, by up-regulating vascular endothelial growth factor (VEGF) and activating the PI3K/AKT signaling cascade, and modulating the innate immune response and epithelialization through their miRNA content (Azevedo et al., 2020; Kasowanjete et al., 2024). Several different types of exosomal microRNA (miRNA) exist, which are involved in regulating various cellular processes, including pro-angiogenesis (upregulation of VEGF) through the PI3K - AKT signaling pathway and promoting the proliferation and migration of endothelial cells through the ERK- MAPK pathway via exosomal protein transfer to endothelial cells. There exists a diverse miRNA content among exosomes that have an immunomodulatory activity, including miR-146a and miR-21, which both downregulate NF-κB signaling and induce M2 macrophage polarization while at the same time reducing the production of pro-inflammatory cytokines (Darlan et al., 2021). Exosomes contain epidermal growth factor (EGF), which promotes keratinocyte growth and stabilization of the epithelial barrier and is thought to mediate these effects via the Wnt/β -catenin signaling pathway (Wang et al., 2014). In combination, these angiogenic, immunomodulatory and pro-epithelial effects enable MSC-Exos to orchestrate a complex signaling network that, in many respects, recapitulates the therapeutic actions of whole-cell MSC therapy. This has positioned MSC-Exos as a promising cell-free approach for the treatment of chronic wounds (Kouroupis et al., 2023). In addition, MSC-Exos may reduce several risks associated with live cell transplantation, including uncontrolled cell proliferation and tumorigenicity. While MSC-Exos have many advantages, there are still many challenges to be addressed before they can be routinely used in clinical practice. Examples of these challenges are the variations in exosome components that can be caused by how the MSC was obtained, the environment where it was cultured, and how the exosomes were separated from the cells, all of which will affect the consistency of their effectiveness for therapeutic use (Mendicino et al., 2014). While large-volume production of exosome products represents a logistical challenge, their bioactivity may be adversely affected by prolonged periods of storage and transportation (Song et al., 2018). In addition, the absence of a sufficient number of standardized potency and dose-response evaluations makes it very difficult to determine the appropriate dose and therefore establish a suitable regulatory framework (Deng et al., 2023).

To successfully navigate and conquer these obstacles, it is necessary to develop and promote standardized methodologies/protocols for isolating exosomes from MSCs so that the exosomal content can be more accurately characterized and agreed upon between regulatory authorities, researchers, and clinicians alike (Chen and Liu, 2016). Once these issues have been resolved, MSC-derived exosomes (MSC-Exos) will be able to proceed from the laboratory to clinic-based care with appropriate dosage forms based on the therapeutic efficacy of MSC-Exos when produced from various sources of MSCs and using different manufacturing techniques.

### 2.5 Fate and effects of MSCs in chronic wounds

Chronic wounds persist in a challenging microenvironment defined by high inflammation, ongoing infection, and combined hypoperfusion/hypoxia and oxidative stress (Zhu et al., 2023; Wang C. et al., 2024). These factors determine the fate of mesenchymal stromal/stem cells (MSCs) for survival, homing, and persistently could affect therapeutic outcome and safety (Zhu et al., 2023; Gao et al., 2024). Evidence suggests that MSC fates in chronic wounds are incumbent upon:

1. Programmed cell death induced by the accumulation of inflammatory cytokines or reactive oxygen species followed by clearance by the immune system which shortens the time MSCs will remain *in situ* and the duration of therapeutic window (Gao et al., 2024; Wang R. et al., 2025);
2. While MSCs could change into fibroblasts, keratinocytes, or endothelial-like cells, the chance of stable long-term integration and functional differentiation occurring in chronic microenvironments is not high, and clinicians rely on more on paracrine and immunomodulatory effects (e.g., angiogenesis, anti-inflammation and epithelialization) (Zhu et al., 2023; Gao et al., 2024);
3. Chronic inflammation and oxidative stress could induce MSC senescence and result in an age-related secretory phenotype thereby impacting repair capacity and increasing local inflammation (Wang B. et al., 2024). Given these characteristics of MSCs, therapeutic approaches should systematically focus around “increasing early survival rates—enhancing paracrine effects—inhibiting senescence—environmental improvement”: routinely debride and control for biofilm/infection prior to delivery, improve perfusion and metabolic support, reduce pro-apoptotic and pro-senescent burden; at the actual delivery level, intra- lesional or peri-lesional local delivery should be highlighted with injectable hydrogels, decellularized scaffolds or scaffold controlled release to maximize time *in situ* and provide controlled release system (Wang C. et al., 2024; Barbon et al., 2025); enhance tolerance and homing via hypoxic or inflammatory “preactivation”, enhancement of the SDF-1α/CXCR4 axis, antioxidant therapy, or metabolic control (Gao et al., 2024; Sajjad et al., 2024); in situations where long-term cell survival is difficult or an infection risk is high, consideration could be given to utilization of MSC-derived extraceullular vesicles (MSC-EVs) or conditioned media as functionally equivalent or complementary solutions, including combinations of material–EV for sustained release and targeted effects (Welsh et al., 2024; Zhu et al., 2025); on the manufacturing and quality level—optimization should occur with early passage and standardization all processes while developing release criteria that include phenotypic, functional, and potency testing to reduce batch-to-batch variability and support predictability (Welsh et al., 2024); together chronic wound therapy should focus on paracrine signaling as the core mechanism, microenvironment restoration as the prerequisite, and biomaterials/drug-delivery design as amplifiers; with staged/multiple delivery used to the extend to avoid excess distancing related to wound burden—which will lead to more stable, reproducible, and sustained clinical benefits overall (Zhu et al., 2023; Wang C. et al., 2024).

## 3 Global advances in mesenchymal stem cell therapy for chronic wounds

### 3.1 Progress in basic resea

#### 3.1.1 Animal models

**FIGURE 3:**
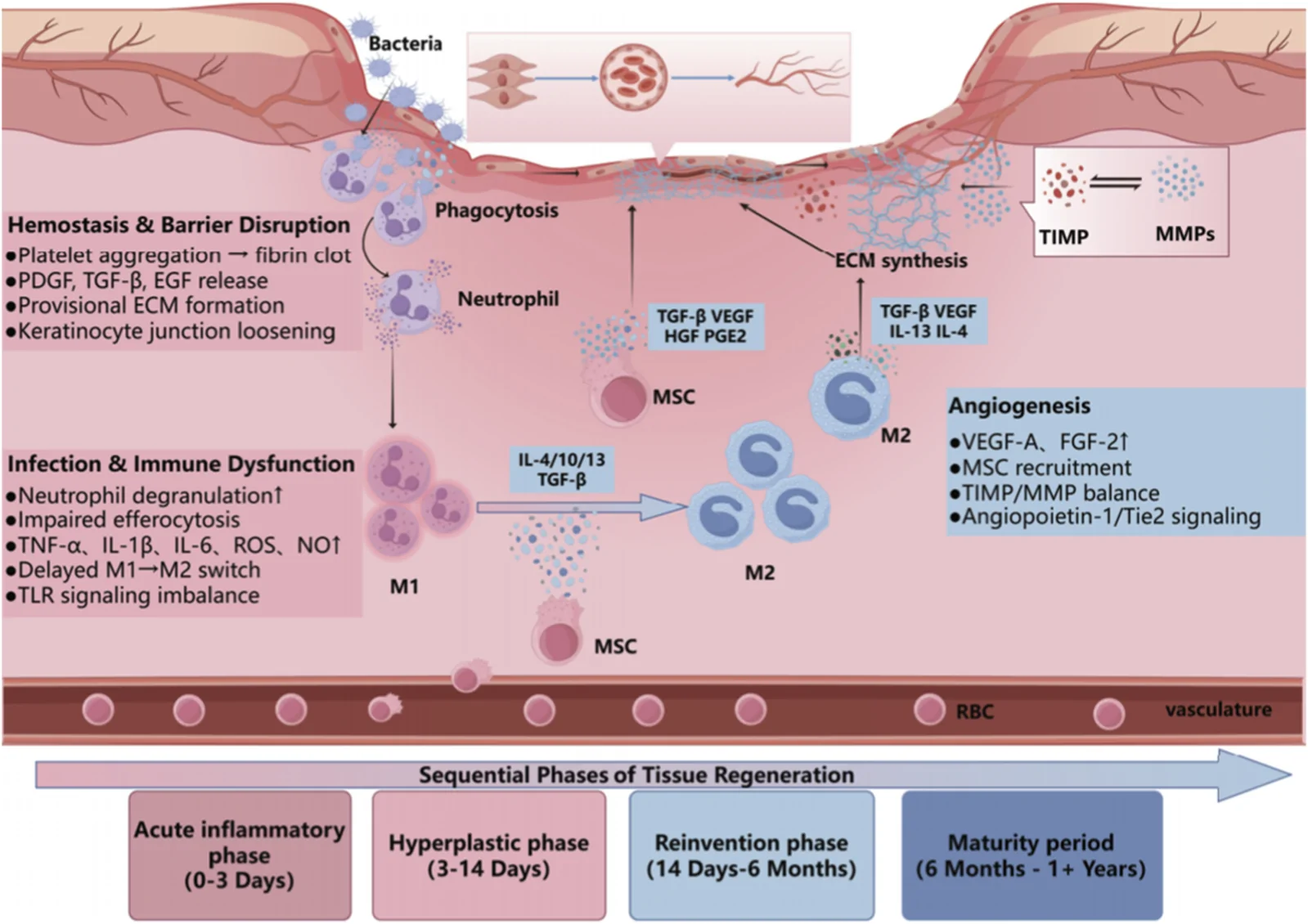
Schematic overview of chronic wound healing phases and MSC-mediated mechanisms. This illustration summarizes the four major stages of chronic wound healing (acute inflammation, hyperplasia, reinvention, and maturation) and highlights the roles of MSCs in modulating immune response, promoting angiogenesis, and supporting ECM remodeling.

Research conducted within experimental animal models provided initial evidence for the potential use of mesenchymal stem cells (MSCs) in wound healing. One early investigation described the ability of these cells to regenerate tissues through multiple mechanisms in preclinical models, leading to significant modality in the literature. In rodent models of diabetic wounds, MSCs supported angiogenesis through the secretion of vascular endothelial growth factors (VEGF) and fibroblast growth factors (FGF); stimulated granulation tissue; and improved wound closure (Azevedo et al., 2020; Song et al., 2018). In addition to promoting the healing of tissue, MSCs reduce the inflammatory environment to promote a decrease in tissue damage through paracrine and/or immunomodulatory pathways. This dual mechanism is particularly evident when assessing als ischemic and inflammatory wounds, where MSCs, in addition to restoring tissue homeostasis, prevented further injury (Zhou et al., 2019). While animal studies provided substantial evidence for the preclinical application of MSCs, there are still barriers to translation, including the source of MSCs, animal models, and route of delivery. For example, MSCs derived from alternate sources (bone marrow, adipose, umbilical cord, etc.) and/or via alternate delivery models (IVM, local injection, etc.) create uncertainty in variable efficacy of the MSCs as a therapeutic strategy. Additionally, genetically engineered MSCs are another option that have been reported to mitigate damage while improving efficacy through the overexpressing VEGF or anti-inflammatory cytokines, however, the long-term stability and safety of a genetically engineered MSC remains a concern (Ezquerra et al., 2021). Even though there are complications in the MSC characteristics, preclinical studies using animal models are compelling to demonstrate the potential of MSCs to regenerate or repair, which will be mirrored to see if the same functional properties define the clinical environment. Future preclinical studies should focus on creating a standardized characterization of animal models, including treatment modalities and outcome evaluations, in addition to continuing to search for models that evaluate long-term safety and functional incorporation of MSCs *in vivo* (Figure 3).

#### 3.1.2 Source-specific properties

The therapeutic efficacy of mesenchymal stem cells (MSCs) is recognized to vary greatly at the original source, with each source experiencing its own advantages and disadvantages. Adipose-derived MSCs are plentiful, easily accessible, and particularly beneficial for repairs of soft tissue or inflammatory processes. However, issues such as donor variability, adipogenic drift, and inconsistent clinical results, continues to persist for these MSCs (Veronesi et al., 2013). Umbilical cord MSCs (UC-MSCs) are highly desirable because of their very favorable immunomodulatory properties, and are further appealing to consider in the context of allogeneic use due to limited immunogenicity (Arthur et al., 2020). Ethical considerations, as well as logistical issues for obtaining and storing umbilical cord derived substrates, are limiting factors as well. Other emerging sources of MSCs that show promise include placenta-derived MSCs and umbilical cord blood MSCs, which share favorable immunological profiles and are not necessarily encumbered with the ethical considerations faced while evaluating UC-MSCs (Saadh et al., 2023); however, limitations associated with the use of these MSCs would remain in relation to availability and development of standardization parameters for the specific clinical applications (Ahn et al., 2021). To better utilize MSC cells and realize their potential in the clinic will require future studies to develop standard protocols for the isolation and characterization of MSCs and potency assays for all sources of MSCs (Al Demour et al., 2021). Additionally, understanding the molecular and functional differences of MSCs relative to specific clinical applications will be critical as well. Finally, developing universal sourcing and characterization protocols will be necessary to ensure consistency of successful outcomes in clinical practice (Table 2).

**TABLE 2:** Comparison of stem cell types used in wound healing.

Stem cell type	Source	Advantages	Disadvantages	Applications in wound healing	References
Mesenchymal Stem Cells (MSC)	Adipose tissue, bone marrow, umbilical cord	Easy to harvest, multipotent, low immunogenicity	Limited regeneration in complex tissues	Chronic wounds, burns, bone fractures, tendon injuries	Sukmana et al. (2023)
AdiposeDerived Stem Cells (ADSC)	Adipose tissue	Readily accessible, high proliferative potential	Limited differentiation in certain conditions	Skin wounds, diabetic foot ulcers, chronic ulcers	Spence and Gallicchio (2022)
Induced Pluripotent Stem Cells (iPSC)	Reprogrammed somatic cells	Pluripotent, ability to generate various cell types	Tumorigenicity, reprogramming inefficiency	Skin wound healing, neural damage repair	Li et al. (2024)
Epithelial Stem Cells (ESC)	Epithelial tissues (skin, corneal)	Ability to regenerate epithelial tissue	Slow proliferation, limited sources	Skin wound healing, neural damage repair	Camoes ˜et al. (2020)

### 3.2 Clinical evidence in chronic wounds

An overview of representative stem cell -based clinical trials and their applications in chronic wound healing is summarized in Table 3. The efficacy of mesenchymal stromal cell (MSC) therapy has progressed through phase I/II studies that report acceptable safety, tolerability, and preliminary benefit for chronic wounds, including diabetic foot ulcers (DFUs), venous leg ulcers (VLUs), and pressure injuries (PIs) (Kishta et al., 2025). The studies highlighted promising findings, but importantly, limitations in the small number of patients, limited time of follow-up, and randomness with which patients are treated, suggest more studies are required in order to implement MSC therapy and support additional use for chronic wounds (Mahmoudvand et al., 2023).

**TABLE 3:** Stem cell-based clinical trials and applications in chronic wound healing.

Disease/target condition	Stem cell type	Delivery approach	Clinical phase	Trial ID	Key results	Safety
Chronic diabetic ulcer	Placenta-derived stem Cells	Local limb injection	I	NCT06373809	Results pending	No serious AEs
Chronic diabetic ulcer	Allogeneic ADSCs	Topical fibrin gel	II	NCT03865394	65% complete healing; 50% wound reduction at 12 weeks	No serious AEs
Chronic diabetic ulcer	Umbilical cord-derived MSC (Corlicyte®)	Ulcer-bed injection in fibrin gel	I	NCT04104451	60% complete healing by day 70 54%–67% wound reduction; increased granulation	No serious AEs
Chronic diabetic ulcer	Adipose-derived MSC	Peri-wound injection	II	NCT02092870	≈50% wound reduction; most wounds healed by12 weeks (expected)	No serious AEs
Chronic diabetic ulcer	Autologous MSC + tibial transport	Tibial transport + local injection	II	NCT05783115	50% wound reduction at 3 months 60% complete healing; blood flow improved in80%	No serious AEs
Chronic venous ulcer	Allogeneic MSC (XSTEM-VLU)	Single local injection	I/II	NCT05549609	≈50% wound reduction by 4 months; 95% healing; ∼70% pain reduction	No serious AEs
Chronic venous ulcer	Umbilical cord-derived MSC	Single local injection	I/IIa	NCT05319106	≈50% wound reduction; ≥95% healing; ∼70% pain reduction	No serious AEs
Chronic venous ulcer	Allogeneic adipose-derived adult MSC	Topical BAMS sheet on fibrin–HA matrix	I/II	NCT05962931	Trial ongoing; efficacy results not yet reported	No serious AEs
Pressure injury	Autologous adipose-derived stromal cells	Local topical/edge application	I	NCT02375802	Pilot protocol; outcomes not yet posted	No adverse Events
Pressure injury	Autologous bone marrow mononuclear cells	Post-debridement infusion	I/II	NCT01572376	Trial completed; results not posted	No serious AEs
Pressure injury	Umbilical cord	Local implantation	II	NCT06302582	Recruiting; no outcomes yet.	No serious AEs

Diabetic Foot Ulcer (DFU): The evidence for the use of MSCs in DFU healing is growing. Wharton’s jelly- derived MSC exosomes were applied topically in a randomized double-blind trial and showed significant improvement in the healing rate, with complete epithelialization seen in a mean of 6 weeks (compared to 20 weeks for the control group) (Kishta et al., 2025). A pilot trial of umbilical cord MSCs, using both perilesional and intravenous administration, showed complete wound closure in a mean of 1.5 months in 80% of patients, with great long-term outcomes and minimal adverse events reported (Zhang et al., 2022). These prior studies are consistent with other studies that highlight the regenerative potential of MSCs for diabetic foot ulcers, suggesting that MSC therapy could reduce time-to-heal and prevent amputations (Sieko et al., 2024).

Venous Leg Ulcer (VLU): For refractory VLUs, autologous bone marrow MSCs were applied in a fibrin spray format and showed a healing rate 10 times higher than the control group, with no serious adverse events reported (Mahmoudvand et al., 2023). Additionally, a phase I/II randomized controlled trial (RCT) is currently underway assessing an allogeneic MSC-seeded biological dressing (BAMS), standardizing biologic off-the-shelf treatment for chronic VLUs. This reflects the increasing interest in cellular therapies for VLU patients (Costela-Ruiz et al., 2025).

Pressure Injuries (PI): Human clinical data for MSCs in PI are limited, but case reports suggest that MSCs (particularly from Wharton’s jelly) show potential for healing when combined with platelet-rich plasma (PRP). One report of a hospital-acquired pressure ulcer showed near-complete healing after 7 weeks of this product being applied, with no adverse events reported (Zhou et al., 2024). Ongoing translational studies utilizing MSC-loaded hydrogels and engineered patches could potentially improve local retention and survival of MSCs in pressure ulcers. However, robust RCTs will be needed to demonstrate efficacy (Barbon et al., 2025).

The important clinical takeaway from above is that mesenchymal stromal cell (MSC)–based interventions are typically used as adjuncts to optimized standard wound care rather than as a replacement for the standard treatment protocol. Most clinical protocols continue to have debridement, infection control, off-loading or compression, vascular optimization, and appropriate dressings as the foundational elements of the management of chronic wounds (Li and Zheng, 2024; Jones et al., 2018), with MSC products serving as biological modulators of the chronic wound environment. While standard management focuses primarily on biomechanical, infectious and ischaemic causative factors for chronic wounds (Sa et al., 2024), MSCs have immunomodulating, pro-angiogenic and pro-regenerative properties (Wu et al., 2024). Thus, MSC- based combination protocols are designed to provide a synergistic or complementary effect with standard management rather than to supplant it (Sukmana et al., 2023). In keeping with this adjunctive approach, emerging research is focused on combining MSCs with other enhanced therapies and biomaterials (Farag et al., 2024; Sukmana et al., 2023). For example, Khalil et al. found that the application of autologous adipose-derived MSCs in platelet-rich fibrin (PRF) with standard care improved rates of ulcer healing compared to PRF alone and raised no significant safety concerns (Khalil et al., 2021). Other reports have identified combinations of MSCs with platelet-rich plasma, fat grafts, dermal substitutes, biological dressings and hydrogel- or scaffold-based delivery systems for DFUs, VLUs and PIs (Xu et al., 2024; Fan et al., 2024). While most of the data available for these applications are preliminary and heterogeneous, collectively they point to the conclusion that MSCs should be considered more realistically as adjunctive and synergistic components of a multimodal approach to wound care rather than as replacements for current evidence-based standard management strategies (Spence and Gallicchio, 2022; Cames et al., 2020).

In summary, early studies show promise for efficacy in chronic wound healing with MSC therapy, and preliminary evidence is emerging. To further establish these findings, additional studies will need to be larger, multi-center RCTs utilizing standardized protocols and interoperable endpoints in order to evaluate the efficacy and safety of MSC therapy for DFU, VLU, and PI patients. Similar to previous commentary, care in determining the precise source of MSCs, route of delivery, and patient selection will ultimately be important to optimizing outcomes in chronic wound care (Zhu et al., 2023).

### 3.3 Technological innovation

The emergence of mesenchymal stromal cells (MSCs) in addition to novel biomaterials and exosomes or exosome-like technologies readily increases their value in regenerative medicine. These techniques are compelling because they address two of the major shortcomings of MSC therapy, namely, poor cell viability in hostile wound niches and variability in therapeutic efficacy, by genetically providing 3D biomimetic microenvironments with controllable local drug delivery and enabling cell-free vesicles to maintain pro-regenerative signalling (Wang D. et al., 2025). However, considerable barriers to translating to clinical efficacies remain including optimization of mechanical properties of biomaterials, durability of *in vivo* biocompatibility and scalable production (Keshavarz et al., 2024).

#### 3.3.1 Differences between 2D and 3D culture for collecting MSC secretome

3D cultures (hydrogels, fibrous/electrospun scaffolds, spheroids or bioreactors) elicit superior ECM signals, cell-to-cell contacts, rigidities like tissues and moderate gradients in oxygen, enabling HIF-1α and mechanotransduction programs that favour a more pro-regenerative MSC secretome and higher quality of EVs (exosomes) than conventional 2D culture systems on rigid plastic (Al-Shaibani, 2022; Sun et al., 2023). Functionally, 3D culture enriches the angiogenic and immunomodulatory cargo and yields EVs with more effects on re-epithelialisation and vascularity than in 2D cultures in wound healing applications (Wu et al., 2018). In this regard silk fibroin gels and exosome-loaded silk fibroin scaffolds are representative 3D matrices which increase survival and adhesion of MSCs, their secretory actions and also allow for the sustained, local delivery of secretomes/EVs (Cheng et al., 2021); alginate/ECM composites further show how 3D microenvironments fine-tune MSC phenotype and paracrine outputs relevant to the repair of wounds (Najafi et al., 2023). Hydrogel-exosome systems have repeatedly shown a more efficacious pro-repair action in chronic wound situations than bolus solutions from flat cultures (Fan et al., 2024). In practice for optimal collection, both 2D and 3D cultures should utilise EV-depleted media or serum- free conditions within a defined conditioning window but 3D systems require an awareness of diffusion and matrix retention; gentle collection and ultrafiltration (size-exclusion chromatography) of exosomes will yield improved exosome recovery and purity and the report of both cell numbers/viability, culture volumes/times, O2 tensions and matrix stiffness will ease batch comparisons (Kim et al., 2022).

Aken together, when the aim is to harvest potent MSC secretome/EVs for therapeutic use, 3D culture is generally preferred over 2D due to higher yield and more pro-regenerative cargo/function.

#### 3.3.2 Biomaterial scaffolds and 3D microenvironments for MSC support

Natural and synthetic scaffolds provide mechanical support and controlled release, while displaying integrin-interacting motifs encompassing MSC adhesion, survival and lineage-based cues (Keshavarz et al., 2024). Silk-fibroin hydrogels and exosome-laden silk-fibroin scaffolds yield supportive 3D niches that yield improved osteogenic/repair outcomes and sustained factor/EV presentation (Cheng et al., 2021; Kim et al., 2021). Alginate-sulfate/ECM composite hydrogels and related bioactive matrices further demonstrate whereby systemic tuning of phenotype and paracrine enhancement are afforded the relevant potential for wound repair (Najafi et al., 2023). Beyond silk-fibroin and alginate systems, therapeutic nanocomponent- containing composites and patient-specific 3D constructed scaffolds are being investigated as a potential means of combining structural support with localized delivery of sustained factors in hard to heal wounds. Hydrogel-exosome “hybrid” systems also protect EV cargo whilst prolonging local bioactivity, to improve re-epithelialization and angiogenesis in chronic wounds (Fan et al., 2024).

#### 3.3.3 Preconditioning strategies that augment MSC potency

This subsection discusses preconditioning strategies—hypoxia, pharmacologic priming (cytokines/small molecules), and physical interventions—to enhance MSC paracrine function and viability before transplantation. Hypoxic preconditioning (∼1–5% O2) activates HIF-1α and increases survival/angiogenic mediators (e.g., VEGF, IGF-1), which enhances MSC persistence, angiogenesis and immune modulation in chronic-wound settings (Mahjoor et al., 2023; Yang Y. et al., 2022). Pharmacologic priming with cytokines (e.g., IFN-γ, TNF-α) or small molecules (e.g., deferoxamine) also improves immunomodulatory capacity and the resistance of MSCs to stress, while workflow types focused on engineering applications yield useful standards for reporting and standardization helpful for translation. Physical cues, such as controlled mechanical loading or photobiomodulation, may cascade with hypoxic/pharmacologic conditioning and additionally increase trophic-factor release and resilience to inflammatory stress (Wang D. et al., 2025). Standardization of EV isolation/characterization and potency metrics is more essential for the translational potential of preconditioned MSC-EV workflows than for clinical applications (Kalluri and LeBleu, 2020; Kim et al., 2022).

## 4 Key challenges and future directions

### 4.1 Long-term safety and standardization research

#### 4.1.1 Long-term safety concerns

Long-term safety associated with MSC-based therapies remains an ongoing issue that limits their broader clinical translation (George et al., 2021). While short-term safety and tolerability outcomes appear relatively consistent across early-phase trials, data on long-term safety endpoints—such as cell persistence, biodistribution and delayed adverse events—are still limited (Mongui-Tortajada et al., 2019). Several important safety issues have been identified in the literature, including tumorigenic potential of engrafted cells, ectopic tissue formation, and immune dysregulation (Squillaro et al., 2016). Certainly, it is obvious that the majority of clinical trials currently being conducted do not track patients beyond the 12–24 months mark, limiting the ability to identify complications that may occur later down the line (Ning et al., 2018). Therefore, long-term studies and international patient registries will be paramount to investigate MSC engraftment, long-term immune modulation effect, and abnormal tissue growth or autoimmunity risk.

(Shi et al., 2025). The approval of new MSC-based therapies, particularly in various areas of therapeutic applications, will be contingent on the development of post-marketing surveillance systems to be transparent and operationally supportive of public trust in their use (Harrell et al., 2019a). The need for long-term data and patient registries to address safety, as well as standardization of MSC preparation and delivery systems will be equally critical if consistent and safe clinical outcome are to be achieved.

#### 4.1.2 Standardization of MSC production

The lack of standardized methods for MSC isolation, expansion, and characterization significantly contributes to variability in outcomes. The biological heterogeneity of different MSC types (e.g., bone marrow, adipose tissue, and umbilical cord) further complicates reproducibility (Chen and Liu, 2016). Additionally, the absence of standardized potency assays to correlate *in vitro* functional characteristics with *in vivo* therapeutic activity results in unpredictability concerning clinical outcomes (Phinney and Pittenger, 2017). Achieving global consensus on MSC production standards—covering aspects such as cell source, purification methods, potency testing, and quality control—is essential for ensuring consistent safety and efficacy (Galipeau and Sens, 2018). Harmonized standards will expedite regulatory evaluation and verification, facilitate reproducibility in clinical applications and studies, and enable broader extrapolation and acceptance of MSC technologies across the healthcare continuum.

### 4.2 Advancement of clinical trials

#### 4.2.1 Multicenter randomized controlled trials (RCTs)

A limitation of current studies involving MSCs has been their small sample size, they are also limited due to having been conducted at only one site (single center), due to relatively short follow-up duration and because they use methods to assess MSCs that differ from one another; this reduces the validity and generalizability of the findings (Harris et al., 2022). Multicenter randomized controlled trials are needed to affirm or validate MSC therapeutic products for specified patient populations, with prespecified, harmonized inclusion and exclusion criteria across centers and stratified randomization to balance key prognostic factors. Furthermore, clinically relevant standardized endpoints—such as complete wound epithelialization at a defined time point, the proportion of ulcers achieving ≥50% area reduction at 12 weeks, recurrence rates at 6–12 months, patient-reported results (e.g., pain and quality of life) and economic measures of cost-effectiveness—should health in future RCTs (Mezey, 2022). Such a core outcome set is essential to provide robust and comparable evidence for the transition of MSC therapies into clinical practice. Future RCTs should also require extended follow-up periods of at least 12–24 months to evaluate the durability of wound closure and to measure long-term safety and efficacy (Baek et al., 2024). Head to head studies comparing various sources of MSCs such as bone marrow, adipose tissue or umbilical cord, different delivery systems (e.g., topical application, peri-wound injection, systemic infusion), and various dosages will provide valuable information for establishing systematic evidence for different types of MSCs and will aid in refining treatment protocols for everyday use in clinical practice (Yin et al., 2016). Without these efforts, the field will develop different datasets that cannot be compared, thereby preventing the transition of MSC therapies into clinical practice settings.

#### 4.2.2 Interdisciplinary collaboration

**FIGURE 4:**
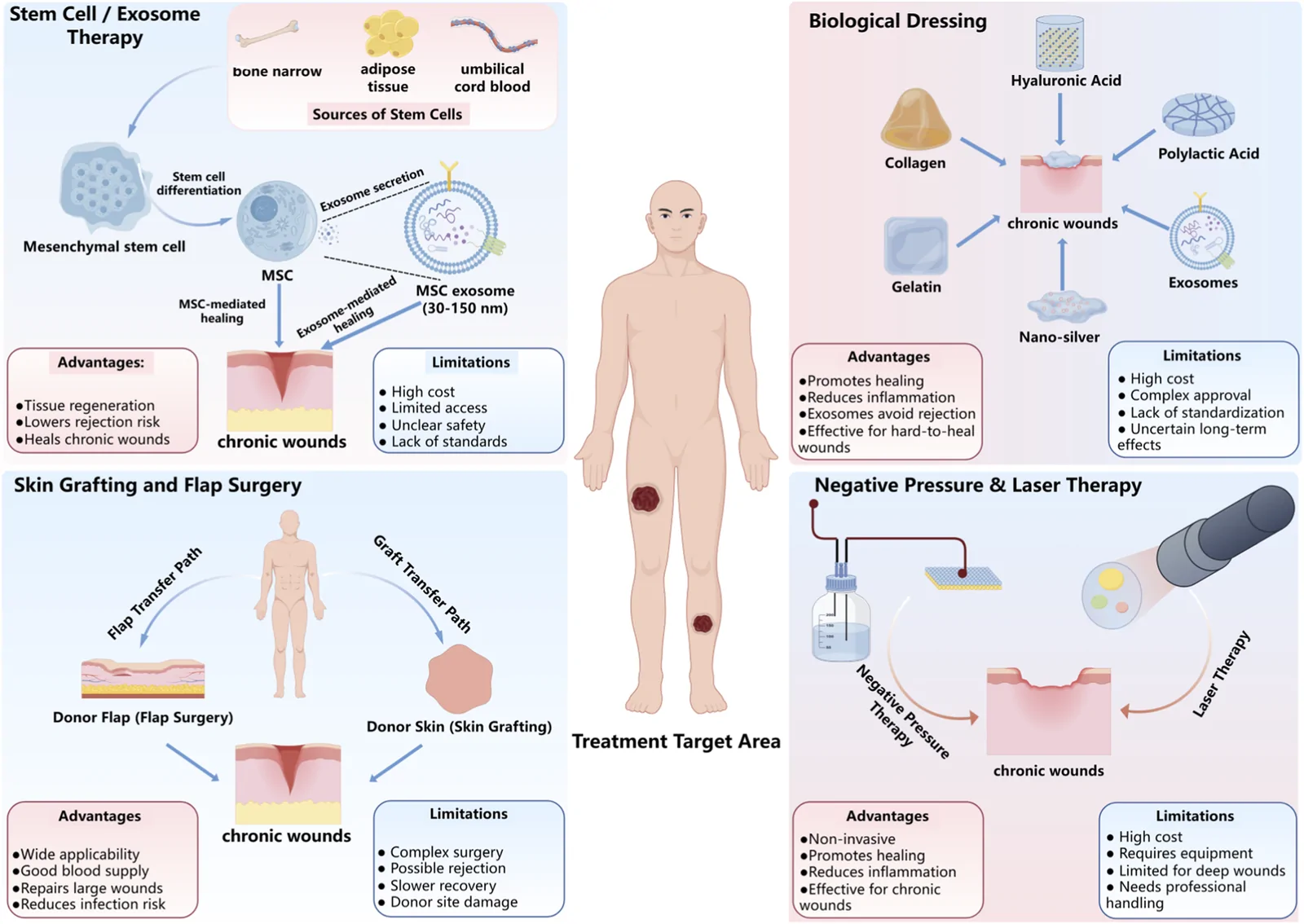
Therapeutic Approaches for Chronic Wound Healing. This figure summarizes key therapeutic strategies for chronic wound healing, including Stem Cell/Exosome Therapy, which uses MSCs and exosomes to promote tissue regeneration and immune modulation; Skin Grafting and Flap Surgery, which involves transferring donor tissue to the wound site for repair; Biological Dressing, such as collagen and hyaluronic acid, which support wound healing and reduce inflammation; and Negative Pressure and Laser Therapy, non-invasive methods that enhance tissue regeneration, blood flow, and reduce infection risk. Each method has distinct advantages and limitations in clinical application.

The successful application of MSC-based therapies requires the collaboration of many disciplines, including, but not limited to, cell biology, biomaterials engineering, clinical practice, and regulatory agencies (Han et al., 2024). During the preclinical period, the collaboration of cell biologists, immunologists and biomaterial engineers will allow for the optimal sourcing of MSCs (mesenchymal stem cells), the application of preconditioning techniques and the development of 3D systems for delivering the cells into a chronic wound environment that promotes cell viability and paracrine signalling. In addition, the input of clinical practitioners and wound-care specialists will be crucial to determining realistic inclusivity criteria for participants in the trial, establishing valid standard-of-care comparison therapies and ensuring that protocols designed for trials can be seamlessly integrated into current wound care protocols. Innovative technologies, such as genetic editing, smart biomaterials, and cell-free exosome therapeutics, introduce new considerations in treatment development that require collaborative integration more than ever, especially for chronic wounds (Farag et al., 2024). In conjunction with the work of data scientists and bioinformaticians, digital wound assessment tools are being developed, as are centralised trial registries and the use of artificial intelligence to assist in the analysis of data that can be used to identify predictive biomarkers, improve patient stratification and guide adaptive trial designs. Health economists and regulatory experts will ensure that the proposed studies consider the cost-effectiveness of a proposed treatment and comply with the requirements for advanced therapy medicinal products (Sukmana et al., 2023). Cross-regional and global research collaboration will enable these parties to harmonize regulatory processes, share data from clinical trials, and thereby continue to examine safety and efficacy (Spence and Gallicchio, 2022). Cross-regional collaboration will also contribute to the standardized manufacture of MSC therapies and regenerative medicine products, which will be scalable, affordable, and follow GMP standards (Li et al., 2024; Cames et al., 2020). Ultimately, such structured, task-oriented international collaboration will be critical to moving MSC therapies from experimental interventions to standardized, clinically validated, affordable, and accessible routines for chronic wound care, as highlighted in Figure 4. In an ideal scenario, these concerted efforts would converge towards internationally accepted, high-level standards of care for chronic wounds. As highlighted in Figure 4, MSC-based therapies must be explored further to address chronic wound care and define how these new technologies will be linked to practice. In a perfect world, for chronic wound care, the concerted efforts of everyone would lead to the creation of standardized Level 1 care for chronic wounds internationally.

## 5 Conclusion

Mesenchymal stem cells (MSCs) represent a paradigm shift in the management of chronic wounds, with their ability to modulate inflammation, promote angiogenesis and facilitate tissue repair through paracrine mechanisms. Mechanisms such as these may help to compensate for the intrinsic shortcomings and limitations of conventional therapies that are unable to meet the complex and multifactorial problem of chronic wounds. Such biological suspension therapies are an enticing biological rationale for their use as additions or adjuncts to conventional therapies, particularly for wounds that are unresponsive to optimized standard care and may benefit from multimodal, synergistic approaches. Despite this promise, numerous challenges still exist for the clinical translation of MSC-based therapies. Source heterogeneity, leading to inconsistent long-term biosafety and expected therapeutic outcomes, and lack of standardization in protocols for preparation and delivery of MSC products create inconsistency in the agreed treatment protocols. Also, the long-term survival of MSCs is compromised in the hostile wound micro-environment, and the severe lack of robust long-term safety data continues to be an impediment to clinical translation and use of MSC therapies. Further, standardization is critical, as standard potency assays, better delivery mechanisms and development of robust manufacturing protocols would be a necessity in translation of MSC therapies to reliable and reproducible clinical practice.

To bring chronic wounds back to long-term health, the following three areas need attention for the field to move forward. First, innovation in technology, particularly in the design of smart biomaterials that can help to enhance survival of MSCs and targeted delivery, the incorporation of artificial intelligence platforms to monitor treatment efficacy and personalize therapeutic approaches, and the use of gene-editing technologies to optimize MSC function, allowing for predictable and enhanced healing responses. Second, regulatory harmonization through globally accepted standards for MSC sourcing, expansion, characterization, and clinical use is urgent to improve the quality of trials, efficacy, and approval pathways. Lastly, long-term safety studies must be prioritized, with an emphasis on robust functional validation and adequate follow-up rates. Long-term safety studies could provide quality measures in establishing the durability of MSC-related healing, noting possible risks for tumorigenicity while monitoring for immune dysregulation. Multi-center, international collaborations and partnerships will be essential in conducting long-term safety studies and in promoting MSC-based therapies to achieve the highest standards in safety for clinical use.

Ultimately, the broader success of translation of MSC-based therapies will depend on cross-sector inter- and multi-disciplinary collaboration in the scientific, clinical, and industrial arenas aligned with educational activities. Partnerships in global contexts will be essential for creating standards for protocols, and for sharing clinical trial and regulatory experience across varied, disparate healthcare systems. This is ultimately the only way to move MSC-based therapies into accepted work practice in clinical management of chronic wounds as adjunctive, synergistic components of established wound-care pathways, where they are already being used with emerging evidence, and beyond into new therapeutic avenues and approaches for chronic complex non-healing wounds.
